# Therapeutic Effect of Curcumin on 5/6Nx Hypertriglyceridemia: Association with the Improvement of Renal Mitochondrial β-Oxidation and Lipid Metabolism in Kidney and Liver

**DOI:** 10.3390/antiox11112195

**Published:** 2022-11-06

**Authors:** Zeltzin Alejandra Ceja-Galicia, Fernando Enrique García-Arroyo, Omar Emiliano Aparicio-Trejo, Mohammed El-Hafidi, Guillermo Gonzaga-Sánchez, Juan Carlos León-Contreras, Rogelio Hernández-Pando, Martha Guevara-Cruz, Armando R. Tovar, Pedro Rojas-Morales, Ana Karina Aranda-Rivera, Laura Gabriela Sánchez-Lozada, Edilia Tapia, José Pedraza-Chaverri

**Affiliations:** 1 Department of Cardio-Renal Physiology, National Institute of Cardiology “Ignacio Chávez”, Mexico City 14080, Mexico; 2 Department of Biology, Faculty of Chemistry, National Autonomous University of Mexico, Mexico City 04510, Mexico; 3 Department of Cardiovascular Biomedicine, National Institute of Cardiology “Ignacio Chávez”, Mexico City 14080, Mexico; 4 Department of Experimental Pathology, National Institute of Medical Science and Nutrition “Salvador Zubirán”, Mexico City 14080, Mexico; 5 Department of Nutrition Physiology, National Institute of Medical Science and Nutrition “Salvador Zubirán”, Mexico City 14080, Mexico

**Keywords:** curcumin, chronic kidney disease (CKD), lipid metabolism, dyslipidemia, mitochondrial dysfunction, liver alteration in CKD, hypertriglyceridemia, fatty acids ß-oxidation, antioxidant, triglycerides

## Abstract

Chronic kidney disease (CKD) prevalence is constantly increasing, and dyslipidemia in this disease is characteristic, favoring cardiovascular events. However, the mechanisms of CKD dyslipidemia are not fully understood. The use of curcumin (CUR) in CKD models such as 5/6 nephrectomy (5/6Nx) has shown multiple beneficial effects, so it has been proposed to correct dyslipidemia without side effects. This work aimed to characterize CUR’s potential therapeutic effect on dyslipidemia and alterations in lipid metabolism and mitochondrial ß-oxidation in the liver and kidney in 5/6Nx. Male Wistar rats were subjected to 5/6Nx and progressed by 4 weeks; meanwhile, CUR (120 mg/kg) was administered for weeks 5 to 8. Our results showed that CUR reversed the increase in liver and kidney damage and hypertriglyceridemia induced by 5/6Nx. CUR also reversed mitochondrial membrane depolarization and β-oxidation disorders in the kidney and the increased lipid uptake and the high levels of proteins involved in fatty acid synthesis in the liver and kidney. CUR also decreased lipogenesis and increased mitochondrial biogenesis markers in the liver. Therefore, we concluded that the therapeutic effect of curcumin on 5/6Nx hypertriglyceridemia is associated with the restoration of renal mitochondrial ß-oxidation and the reduction in lipid synthesis and uptake in the kidneys and liver.

## 1. Introduction

Chronic kidney disease (CKD) has an incidence of over 11% worldwide [1], making it one of the most important diseases on the earth. CKD is characterized by different symptoms such as a decrease in the glomerular filtration rate to less than 60 mL/min per 1.73 m^2^ for 3 months or more, an increase in plasma nitrogenous compounds (creatinine and urea), hypertension, and dyslipidemia [2]. Dyslipidemia is characterized by high concentrations of plasma lipids, mainly triglycerides (TG) and cholesterol (CH), which are related to their transport proteins called lipoproteins [3]. These specialized macrostructures are agglomerations of lipids and proteins responsible for collecting and distributing lipids in the body. Their name is based on their density, with very low-density lipoproteins (VLDL) and low-density lipoproteins (LDL) responsible for distributing triglycerides mainly; meanwhile, high-density lipoproteins (HDL) are responsible for collecting cholesterol and taking it back to the liver [4]. In CKD, dyslipidemia is associated with the formation of atheroma that leads to cardiovascular diseases (CVD) and the accumulation of lipids in parenchymal organs, causing their malfunction [5,6].

In CKD, VLDL and LDL plasma levels are elevated, and HDL concentrations are decreased, causing TG and CH [7,8] plasma accumulation. Concerning this, it has been seen that 10% of patients with CKD die due to CVD derived from dyslipidemia. Likewise, the presence of a fatty liver and the accumulation of lipids in the kidney have been observed [9]. However, the molecular mechanisms that cause dyslipidemia in CKD are poorly understood, and their relationship with renal and hepatic metabolism alterations is still under study.

One of the most used models for the CKD study is the 5/6 nephrectomy (5/6Nx). This animal model is characterized by the elimination of 5/6 parts of the renal mass, causing maladaptive changes that trigger CKD [10,11,12], which includes dyslipidemia that is present even if the diet is controlled and balanced [13,14,15]. Although CKD mechanisms induced by 5/6Nx have been extensively studied, most works have been focused on renal or cardiac hemodynamic alterations [11,16,17]. However, until now, the mechanism involved in dyslipidemia development in peripheral tissue and how this affects the kidney is not well understood and has been poorly explored. It is known that in CKD, the levels of fatty acid synthesis proteins increase in the liver, particularly fatty acid synthase (FAS) and acetyl CoA carboxylase (ACC) [14].

Meanwhile, there is a decrease in carnitine palmitoyl transferase 1 (CPT1), adenosine triphosphate (ATP) synthase α and β levels, suggesting a possible β-oxidation reduction [14]. Likewise, it decreases the hepatic accumulation of triglycerides and the proteins related to their formation (diacyl glycerol acyl transferases 1 and 2 or DGAT1 and DGAT2) [14]. In the case of the kidney, it was observed that mitochondrial biogenesis decreases, as well as the impairment in mitochondria shape and function and the oxygen consumption decrease associated with lower ATP formation [18]. However, these potential mitochondrial alterations in 5/6Nx-induced CKD have not been described in the liver, so it is unknown whether the mitochondrial function is also altered in this organ. On the other hand, it is not known if changes in lipid metabolism also affect the concentration of the different fatty acids enzymes that have activity in liver and kidney tissue.

To correct dyslipidemia in patients with CKD, fibrates or statins that increase HDL and decrease LDL, respectively, are commonly used. However, both drugs cannot be used simultaneously or for long time periods because they cause myopathies to delay the patient’s recovery [19,20]. Currently, using natural products without side effects has been studied as a palliative in the treatment of dyslipidemia. Among them, one of the most promising is curcumin, a pigment extracted from the *Curcuma longa* plant that has shown multiple beneficial effects. The most prominent is its antioxidant power, which has also been related to the capacity to reduce lipids levels [21,22]. The effect of curcumin on dyslipidemia has been extensively evaluated in models of diabetes and obesity, proving its beneficial and corrective effect. In these models, curcumin decreases serum and hepatic concentrations of TG, CH, free fatty acids (FFA), and LDL [22]. It also increases the serum concentrations of HDL and the activity of various lipases that promote the entry of lipids into the tissues [22]. However, it is still unknown if curcumin has protective effects on the lipid levels in CKD, because the pathophysiology of dyslipidemia may be completely different. 

In this context, in the 5/6Nx model, the treatment with 75 mg/kg of curcumin for 10 weeks, starting one-week post-surgery decreases the LDL and cholesterol concentrations [23]. Likewise, in the CKD model induced by adenine (0.75%), curcumin treatment with 50, 100, and 150 mg/kg for 24 days, since the beginning of the protocol, corrected the serum lipid profile, increased HDL cholesterol (HDLc) concentration, and decreased CH, TG, LDLc, VLDLc concentrations [24]. In addition, they found [24] that curcumin decreased non-esterified fatty acids (NEFA), and the atherogenic index and the coronary risk index. In the liver, increased CH and decreased TG and NEFA concentrations were observed [24]. However, in the previous studies [23,24], curcumin administration started at the same time as adenine administration [24] or two weeks after the surgery to establish CKD, so these treatments can be considered a preventive protocol, which avoids the progression of acute kidney injury to CKD. Thus, it is still unknown if curcumin can be used as a corrective treatment for CKD hyperlipidemia. Furthermore, more studies are still necessary to elucidate the molecular mechanisms by which curcumin could correct dyslipidemia, particularly in the kidney and liver. The study aimed to evaluate the potential therapeutic effect of curcumin on 5/6Nx dyslipidemia and the effect of this antioxidant on kidney and liver lipid metabolism.

## 2. Materials and Methods

### 2.1. Reagents

Curcumin (C1386) and anti-4-hydroxynonenal (4-HNE, ab5605) antibodies were purchased from Sigma-Aldrich Chemical Co (St. Louis, MO, USA). Plasma creatinine and triglyceride concentrations were measured using commercial kits from Spinreact (Girona, Spain). Plasma concentrations of total CH, LDLc, and HDLc were analyzed by using enzymatic colorimetric kits (Roche Diagnostic; Indianapolis, IN, USA) Anti-kidney injury molecule-1 (Kim-1, AF3689) antibodies were purchased from Bio-Techne (Minneapolis, MN, USA). Anti-ATP synthase 5a (ATP5a 7H10BD4F9) antibodies were purchased from Thermo Fisher (Waltham, MA, USA). Anti-proliferator-activated receptor gamma (PPARγ, ab272718) antibodies and anti-peroxisome proliferator-activated receptor alpha (PPARα, ab227074) antibodies were purchased from Abcam (Cambridge, UK). Anti-ACC (GTX132081) antibodies, anti-FAS (GTX109833) antibodies, anti-β-actin (GTX109639) antibodies, anti-diacylglycerol acyl transferase 1 (DGAT, GTX48577) antibodies, anti-anti-sterol receptor element binding protein 1 (SREBP1, GTX79299) antibodies were purchased from Genetex (San Diego, CA, USA). Non-fat dry milk and anti-peroxisome proliferator-activated receptor γ co-activator 1 α (PGC1α, sc-518025) antibodies, anti-voltage-dependent anion channel (VDAC, sc-390996) antibodies, and anti-transforming growth factor beta (TGFβ, sc-130348) antibodies were purchased from Santa Cruz Biotechnology (Dallas, TX, USA). Anti-CPT1, (D3B3) antibodies, anti-cluster of differentiation 36 (CD36, 14347) antibodies, anti-rabbit IgG horse radish peroxidase (HRP) linked antibody (7074S), and anti-mouse

### 2.2. Experimental Protocol 

**Figure 1:**
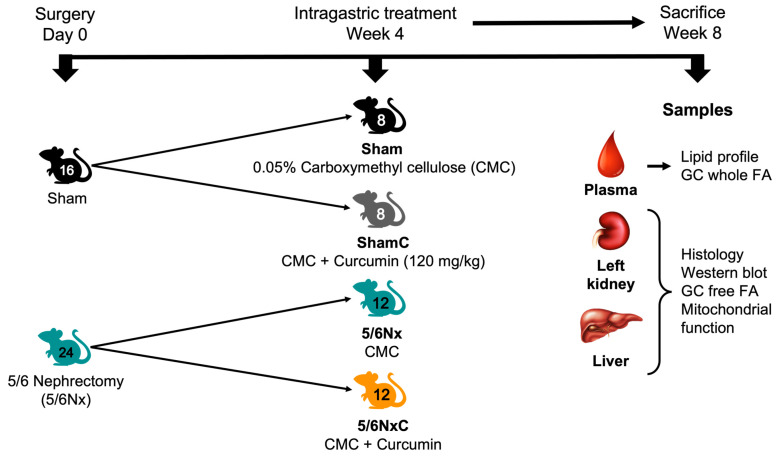
Experimental design. GC = Gas Chromatography and FA = Fatty Acids.

Forty male Wistar rats weighing 230 to 250 g, from the animal facility of the National Institute of Cardiology “Ignacio Chávez” (Mexico City) were used. The Institutional Animal Care and Use Committee (CICUAL) approved the experimental protocol under the number INC/CICUAL/013/2022. To perform the surgeries, the animals were first divided into two groups: Control (Sham) which underwent exploratory surgery, and 5/6Nx, where a right unilateral nephrectomy was performed, and 2/3 parts of the renal arteries of the left kidney were obliterated to maintain functional 1/6 part of the total renal mass [11,12,17]. The animals were allowed to develop CKD with food and water ad libitum for four weeks. At the end of the four weeks, the gavage treatments began. The animals were again divided into four groups: Sham, treated with 0.05% carboxymethyl cellulose (CMC); Sham curcumin (ShamC), treated with CMC and curcumin at 120 mg/kg; 5/6Nx treated with CMC and 5/6Nx curcumin (5/6NxC) treated with CMC and curcumin for another four weeks. The animals were sacrificed at week 8, and plasma, liver, and remnant kidney were collected (Figure 1).

### 2.3. General Parameters

The amount of food and water consumed and the animals’ weight, were recorded throughout the last week of the experiment. 

### 2.4. Plasma Lipid Profile

Plasma concentrations of total CH, LDLc, and HDLc were analyzed using the Cobas C111 analyzer (Roche Diagnostic; Indianapolis, IN, USA). The concentrations of VLDLt were estimated using Friedewald´s formula (VLDLt = triglycerides/5), and VLDLc was obtained from the formula: VLDLc = CH-(LDLc + HDLc) [25].

### 2.5. Gas Chromatography

The fatty acid extraction was carried out under Folch´s method [26]; 100 µL of plasma or homogenate tissue and 50 µL of a known concentration of heptadecanoic acid (C17:0) as an internal standard was used. First, the samples were extracted with chloroform/methanol (2:1) mixture and centrifugation for two min two times. After centrifugation, the organic layer was collected, dehydrated with sodium sulfate, filtrated, and then evaporated at 36 °C under a constant nitrogen stream. Immediately, the derivation was carried out with methanol, 2% H_2_SO_4_ and toluene at 90 ºC for 2 h for plasma total fatty acids; for tissue FFA methanol, 2% H_2_SO_4_ and 2,2-methoxypropane at 23 °C for 15 min were used. Finally, fatty acid methyl esters were extracted with hexane/5% sodium chloride (2:1) mixture two times, and the superior phase was collected and evaporated with a nitrogen stream. The dry residue was dissolved in hexane (50 μL), and 1 μL was used for the analysis in a Shimadzu gas chromatographic system (Shimadzu, Kyoto, Japan) with flame detection on a capillary column. All solvents and chemicals were analytical grades from J. T Baker (Avantor Performance Materials, Central Valley, PA, USA). The data were adjusted to molar concentration and normalized with total protein for tissue samples [27].

### 2.6. Histology

Thin slices were obtained from sagittal kidney and liver sections and immediately fixed by immersion in buffered formalin (pH 7.4), dehydrated and embedded in paraffin. To evaluate histological damage, the kidney and liver sections were stained with hematoxylin/eosin. Other sections from the kidney were stained with Masson trichrome and used to determine fibrosis in a blinded fashion. The total area of the kidney was measured by automated morphometry, and the blue stained areas (excluding glomeruli and vessels) that corresponded to fibrosis in the cortex interstitial compartment were measured, and its percentage was determined. Fragments of kidney and liver tissues were frozen by immersion in liquid nitrogen immediately after euthanasia, and frozen sections were obtained with a cryostat and stained with red oil to determine lipid deposits. 

### 2.7. Western Blot

To determine expression patterns of different proteins, kidney and liver tissues were homogenized in RIPA buffer containing proteases inhibitors. Then, 30 µg of protein was loaded onto a 10% SDS-Polyacrylamide gel and electrophoresis was conducted for 2 h (Mini Protean, Bio-Rad, Hercules, CA, USA). After that, proteins were transferred to a nitrocellulose membrane at 100 volts for 35 min using a Criterion Blotter device (Bio-Rad, Hercules, CA, USA). Membranes were stained with Ponceau red to confirm protein transfer. Subsequently, membranes were washed and blocked with non-fat dry milk in tris buffed saline (TBS)-Tween buffer for 1 h. Primary antibodies were incubated overnight at 4 °C. The antibodies used were as follows: Kim-1 (1:2000 dilution), PPARγ (1:5000 dilution), DGAT (1:5000 dilution), CD36 (1:10,000 dilution), ACC (1:3000 dilution), FAS (1:4000 dilution), PPARα (1:5000 dilution), PGC1α (1:3000 dilution), ATP5a (1:20,000 dilution), CPT1 (1:7500 dilution), SREBP1 (1:5000 dilution), 4-HNE (1:5000), VDAC (1:5000), β-actin (1:10,000 dilution), TGFβ (1:5000 dilution). Anti-rabbit IgG and anti-mouse-IgG secondary antibodies were incubated for two hours at room temperature. Chemiluminescence was determined with a commercial kit (Amersham ECL western blotting detection kit, Buckinghamshire, UK).

### 2.8. Mitochondrial Isolation 

Renal mitochondria were isolated from remnant renal mas by differential centrifugation using the protocol previously described [18]. Briefly, renal tissues were cooled by immersion in isolation buffer A [225 mM D-mannitol, 75 mM sucrose, 1 mM ethylenediaminetetraacetic acid (EDTA), 5 mM 4-(2-hydroxyethyl)piperazin-1-ylethanesulfonic acid (HEPES), 0.1% fatty acids (FA)-free bovine serum albumin (BSA), pH 7.4] at 4 °C and then cut into small pieces. The tissues were homogenized in a glass Potter–Elvehjem with a TeflonVR pestle in the same buffer, and the mitochondria fraction was obtained by differential centrifugation, the final mitochondrial pellet was resuspended in 200 μL of BSA-free isolation buffer, and total protein was determined by the Lowry method [18].

### 2.9. Mitochondrial β-Oxidation Oxygen Consumption and Membrane Potential (ΔΨm)

Oxygen consumption by the mitochondria fraction was performed using a high-resolution respirometer (Oxygraph O2k, OROBOROS, Innsbruck, Austria) at 37 °C. The isolated fraction was loaded into a 2 mL chamber with respiration buffer MiR05 (0.5 mM ethyleneglycol- bis(β-aminoethyl)-N,N,Nʹ,Nʹ-tetraacetic acid (EGTA), 3 mM MgCl_2_, 60 mM K-lactobionate, 20 mM taurine, 10 mM KH_2_PO_4_, 20 mM HEPES, 110 mM sucrose, and 1 g/L essentially FA free BSA, pH = 7.4). Electron transport was started by β-oxidation linked substrates (2 mM L-carnitine, 2 μM palmitoyl-L-carnitine plus 2 mM malate) [28] and state 3 (S3) was stimulated by the addition of 2.5 mM adenosine diphosphate (ADP), meanwhile state 4 was induced by oligomycin (S4o) by the addition of 2.5 μM oligomycin. All parameters were corrected by residual respiration (ROX) values obtained by adding 0.5 µM rotenone plus 2.5 µM antimycin and normalized by protein content. Respiratory control (RC) was defined as the S3/S4o ratio, and OXPHOS-associated respiration (P) was defined as S3-S4o [28].

The changes in mitochondrial membrane potential (ΔΨm) were determined in an O2k-Fluorometer (OROBOROS, Innsbruck, Austria) using safranin O (5 µM) as a probe, as previously reported [29]. Briefly, the changes determine the ΔΨm. To stimulate β-oxidation, the respective substrates (2 mM L-carnitine, 2 μM palmitoyl-L-carnitine plus 2 mM malate) were added. ΔΨm in S3 was obtained by the addition of 2.5 mM ADP and in S4o by 2.5 μM oligomycin, 5 μM carbonyl cyanide 3-chlorophenylhydrazone (CCCP) was added to dissipate the ΔΨm completely and to correct the non-specific interactions. Results were expressed as the changes in the measurable concentration of safranin O solution (ΔμM of Saf) in S3 or S4o concerning CCCP decoupling.

### 2.10. Statistics

Data were reported as mean ± standard deviation (SD). Data were analyzed by one-way ANOVA followed by Tukey’s test for n > 3 and Fisher’s LSD test for n = 3. Differences at *p* < 0.05 were considered statistically significant.

## 3. Results

### 3.1. General Parameters

The water consumption in the animals with nephrectomy was significantly higher than in the Sham groups, but there were no changes with the curcumin treatment (Table 1). On the other hand, the food consumption in the 5/6NxC group was significantly higher than in the 5/6Nx group, which ate less than the Sham and ShamC groups (Table 1). The body weight of the 5/6Nx and 5/6NxC groups was significantly lower than the Sham and ShamC groups, and the 5/6NxC group weighed more than the 5/6Nx group (Table 1).

### 3.2. Kidney and Liver Damage Markers

**Figure 2:**
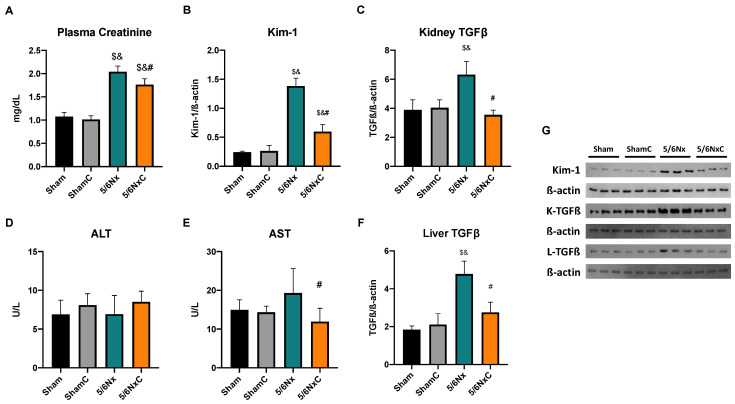
Kidney and liver damage markers in the four groups of rats studied: Sham, Sham + curcumin (ShamC), five-sixths nephrectomy (5/6Nx), and 5/6Nx + curcumin (5/6NxC). (**A**) Plasma creatinine (n = 11), (**B**) Kidney injury molecule-1 (Kim-1, n = 3), (**C**) Kidney transforming growth factor beta (TGFβ, n = 3), (**D**) Plasma alanine aminotransferase (ALT, n = 11), (**E**) Plasma aspartate aminotransferase (AST, n = 11), (**F**) Liver TGFβ (n = 3) and (**G**) representative images of western blot. β-actin was used as a loading control. One-way analysis of variance (ANOVA), post hoc Tukey, Mean ± SD. ^$^ *p* < 0.05 vs. Sham, ^&^ *p* < 0.05 vs. ShamC, ^#^ *p* < 0.05 vs. 5/6Nx. K-TGFβ = kidney TGFβ and L-TGFβ = liver TGFβ.

To assess renal damage in the 5/6Nx and their improvement with curcumin, we evaluated kidney function marker creatinine. We found that the 5/6Nx and 5/6NxC groups presented significantly higher creatinine levels than the Sham and ShamC groups. However, curcumin (5/6NxC) treatment significantly reduced creatinine levels compared to the 5/6Nx group (Figure 2A). In addition, we determined the protein levels of Kim-1 (Figure 2B,G), a marker of proximal tubule damage. We observed that the abundance of this protein was significantly increased in the 5/6Nx group; meanwhile, curcumin treatment significantly decreased it (Figure 2B,G). TGFβ, a fibrosis marker characteristic of CKD, was also measured. The abundance of this protein in the kidney was significantly higher in the 5/6Nx group compared with any other group but decreased significantly in the 5/6NxC group (Figure 2C,G).

In order to determine the presence of liver damage, alanine aminotransferase (ALT) and aspartate aminotransferase (AST) were measured. ALT shows no significant changes in any group (Figure 2D). Meanwhile, AST significantly increased in the 5/6Nx group, and the curcumin treatment decreased it (5/6NxC group) (Figure 2E). Furthermore, liver TGFβ abundance was significantly higher in the 5/6Nx group compared with other groups and decreased significantly in the 5/6NxC group (Figure 2F,G).

### 3.3. Kidney and Liver Histology

**Figure 3:**
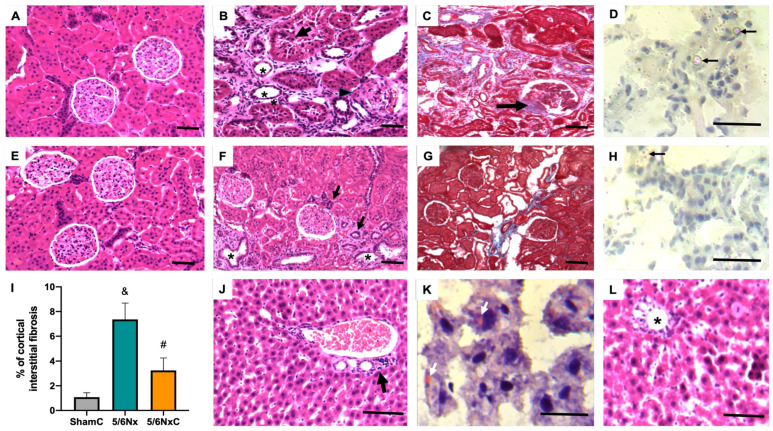
Representative micrographs from the four experimental groups: Sham, Sham + curcumin (ShamC), five-sixths nephrectomy (5/6Nx), and 5/6Nx + curcumin (5/6NxC). (**A**) Histologically normal kidney from Sham group rat. (**B**) In comparison, the 5/6Nx rat kidney shows interstitial fibrosis with chronic inflammatory infiltrate, tubules with atrophic (asterisks) or necrotic (arrow) epithelium, glomerulus with retracted capillaries, and vascular pole fibrosis (arrowhead). (**C**) Masson’s trichrome stain in 5/6Nx rat kidney shows more evident fibrosis, collagen stained blue, particularly in the interstitium and in the vascular pole of some glomeruli (arrow). (**D**) Oil red staining of the 5/6Nx group shows numerous cytoplasmic small red vacuoles in tubular cells that correspond to lipids; some tubules have big lipid drops in the tubular lumen (arrows). (**E**) Kidney section of a ShamC does not show histological alterations. (**F**) Treatment with curcumin in animals with 5/6Nx (5/6NxC group) induces a decrease in renal fibrosis and preservation of cortical tubules, some tubules show necrotic epithelium (asterisks), and others are small and lined with cubic cells with hyperchromatic nuclei corresponding to cells in regeneration (arrows), the glomeruli do not show fibrosis or retraction of the capillaries. (**G**) Masson’s staining shows decreased interstitial fibrosis and glomerular affectation in the 5/6NxC rat kidney. (**H**) Oil red staining in 5/6NxC rat kidney shows some epithelial cells with occasional cytoplasmic lipid vacuoles (arrow). (**I**) The automated morphometric analysis showed a significant decrease in interstitial fibrosis in the 5/6NxC group compared to the 5/6Nx group. (**J**) Portal area of the 5/6Nx liver showing chronic inflammatory infiltrate (arrow). (**K**) Oil red staining shows some 5/6Nx hepatocytes with scare cytoplasmic lipid vacuoles (white arrows). (**L**) The portal areas (asterisk) of the 5/6NxC rat liver without inflammation, some hepatocytes show regenerative changes such as increased cell and nuclear size, hyperchromasia and binucleation. Kidney sections staining H/E and Masson 200× magnification, liver micrographs H/E staining 400× magnification, frozen sections red oil staining 1000× magnification. Bars represent 100 μ in paraffin sections and 50 µ in frozen sections.

The renal histological study of the 5/6Nx groups showed interstitial fibrosis with chronic inflammatory infiltrate; interstitial arterioles showed a thickened and hyalinized middle muscular layer. Numerous glomeruli showed retraction of the capillary tuft with fibrosis that in some glomeruli formed nodules near or in the vascular pole (Figure 3B), which is better evidenced in sections stained with Masson’s trichrome (Figure 3C). Numerous proximal tubules show necrotic epithelium or marked atrophy with enlarged lumens, which are occupied by hyaline cylinders in some tubules. The basement membranes are thickened and have a hyaline appearance. Oil red staining of the 5/6Nx group showed numerous small lipid vacuoles in the cytoplasm of damaged tubular epithelium, and some proximal tubules exhibited large lipid drops that occupied the tubular lumen (Figure 3D). The liver of this group generally shows normal histology; only some portal areas have mild chronic inflammatory infiltrate, and oil red staining showed occasional hepatocytes with scarce small lipid vacuoles in the cytoplasm (Figure 3J,K). The kidneys of the 5/6Nx group treated with curcumin (5/6NxC group) showed a marked decrease in all these histological alterations. There are numerous proximal tubules with regenerative changes in the epithelium and occasional glomeruli with scant fibrosis; automated morphometry showed a significant decrease in interstitial fibrosis (Figure 3I). Oil red staining showed occasional and small lipid vacuoles in some tubular epithelial cells without lipid drops in the tubular lumen (Figure 3F–H), while liver histology does not show portal inflammation but in some areas, there are changes indicative of liver regeneration such as large hyperchromatic nuclei and binucleated hepatocytes (5/6NxC group, Figure 3L), and there were no cytoplasmic lipid vacuoles (5/6NxC group, data not shown). The Sham and ShamC groups show no histological changes in the kidney (Figure 3A,E, respectively) or liver (not shown).

### 3.4. Plasma Lipid Profile

The plasma lipid profile was measured in all the studied groups (Table 2). We observed that TG concentration increased significantly in the 5/6Nx group; this effect was significantly decreased with curcumin treatment (5/6NxC group). In the case of total cholesterol, HDLc and LDLc, the nephrectomy groups were significantly higher than the control groups, but no effect of curcumin treatment was observed. The concentrations of VLDLc did not have significant changes in any group. In contrast, VLDLt increased significantly in the 5/6Nx group compared to the ShamC group and decreased significantly in the 5/6NxC group compared to the 5/6Nx group. 

#### Plasma Fatty Acids Profile

Individual fatty acids were measured by gas chromatography (Table 3) to find out if there was any fatty acid whose plasma concentration changed due to the nephrectomy. In addition, total, saturated, unsaturated, and polyunsaturated fatty acids were calculated (Table 3). Fatty acids were significantly higher in the 5/6Nx group compared to the Sham groups. The fatty acids that significantly increased in the 5/6Nx group concerning the Sham were C16, C16:1n-7, C18, C18:1n-9, C18:2n-6, and C20 and for the 5/6NxC group were C16, C16:1n-7, C18, C18:2n-6, and C20. In the case of C12, its concentrations decreased in the 5/6NxC group compared to ShamC. Curcumin treatment influenced C16:1n-7 concentration which decreased significantly in the 5/6NxC group compared to the 5/6Nx group. On the other hand, saturated fatty acids significantly increased in the nephrectomy groups, and curcumin treatment significantly decreased unsaturated fatty acids compared to the 5/6Nx group (Table 3).

### 3.5. Kidney Lipid Metabolism

#### 3.5.1. The Kidney Free Fatty Acid Profile

To determine if free fatty acids were also changed in kidney tissue, individual FAs were measured (Table S1). In addition, total, saturated, unsaturated, and polyunsaturated fatty acids were calculated (Table S1). Total fatty acids are significantly increased in the 5/6Nx group, particularly the concentration of C16:1n-7 and saturated fatty acids. On the other hand, C12 and C16:1n-7 were increased in the 5/6NxC group compared to the Sham groups (Table S1).

#### 3.5.2. Renal Levels of Proteins Involved in Lipid Synthesis

**Figure 4:**
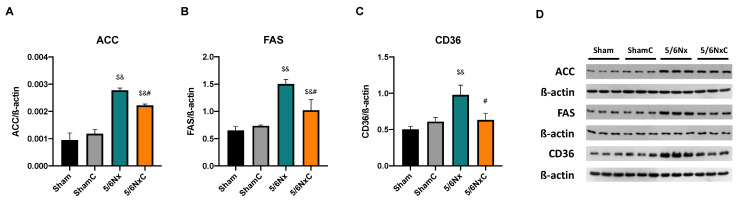
Kidney fatty acids synthesis and storage-related protein abundance in the four groups of rats studied: Sham, Sham + curcumin (ShamC), five-sixths nephrectomy (5/6Nx), and 5/6Nx + curcumin (5/6NxC). (**A**) Acetyl CoA carboxylase (ACC), (**B**) Fatty acid synthase (FAS). (**C**) Fatty acid receptor cluster of differentiation 36 (CD36), and (**D**) representative images of western blot. β-actin was used as a loading control. One-way analysis of variance (ANOVA), posthoc Fisher´s Least significant difference (LSD), Mean ± SD, n = 3, ^$^ *p* < 0.05 vs. Sham, ^&^ *p* < 0.05 vs. ShamC, ^#^ *p* < 0.05 vs. 5/6Nx.

In order to know if fatty acid synthesis was enhanced in kidney and liver tissues, we measured the intracellular levels of ACC and FAS, proteins involved in acetyl-CoA carboxylation to malonyl-CoA and palmitate synthesis from acetyl-CoA and malonyl-CoA. The levels of both proteins were significantly increased in the 5/6Nx group and significantly decreased with curcumin (5/6NxC group) (Figure 4A,B,D). In the case of SREBP1 (Figure S1A), a transcription factor that plays a key role in the induction of lipogenesis, its abundance was significantly increased in the 5/6Nx group, but curcumin treatment did not have any effect on the 5/6NxC group. We further determined the levels of lipid membrane transport CD36 (Figure 4C,D) and found that the protein levels significantly increased in the model, and curcumin treatment significantly reduced it. 

#### 3.5.3. Kidney Mitochondrial β-Oxidation

**Figure 5:**
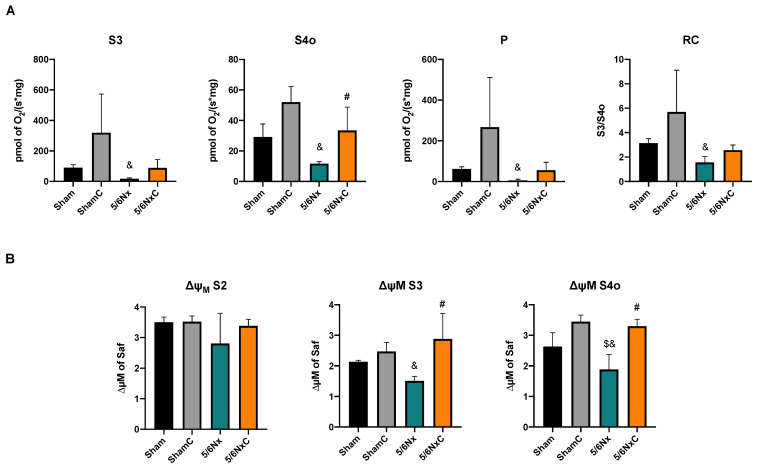
Kidney mitochondrial β-oxidation in the four groups of rats studied: Sham, Sham + curcumin (ShamC), five-sixths nephrectomy (5/6Nx), and 5/6Nx + curcumin (5/6NxC). (**A**) Respiration and (**B**) Membrane potential. S3 = State 3, S4o = State 4 induced by oligomycin, P = Oxidative phosphorylation associated respiration, RC = Respiratory control, ΔΨm = Changes in mitochondrial membrane potential, Safranin (Saf). One-way analysis of variance (ANOVA), post hoc Fisher´s Least significant difference (LSD). Mean ± SD, n = 3. ^$^ *p* < 0.05 vs. Sham, ^&^ *p* < 0.05 vs. ShamC, ^#^ *p* < 0.05 vs. 5/6Nx.

Fatty acid metabolism involves a balance between FA synthesis and degradation. In addition, the ATP production in the proximal tubule depends on fatty acid β-oxidation [30,31]. Thus, we evaluated the mitochondrial respiratory parameters related to β-oxidation. We found that 5/6Nx decreased mitochondrial respiration, and curcumin reestablished it in state 4 (associated with a leak) (Figure 5A). Moreover, curcumin prevented 5/6Nx-induced mitochondrial membrane depolarization in state 3 (state associated with ATP synthesis) and in state 4 induced by oligomycin (Figure 5B), suggesting that curcumin partially restores 5/6Nx-induced mitochondrial depolarization and the capacity of oxidative phosphorylation associated with the fatty acid β-oxidation.

#### 3.5.4. Renal Levels of Proteins Involved in Biogenesis and FA Transport in Mitochondria

**Figure 6:**
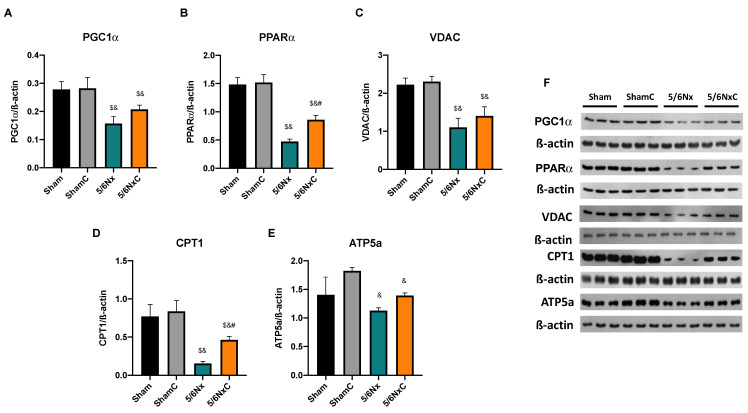
Kidney mitochondrial biogenesis and β-oxidation related proteins abundance in the four groups of rats studied: Sham, Sham + curcumin (ShamC), five-sixths nephrectomy (5/6Nx), and 5/6Nx + curcumin (5/6NxC). (**A**) Peroxisome proliferator-activated receptor γ co-activator 1α (PGC1α), (**B**) Peroxisome proliferator-activated receptor alpha (PPARα), (**C**) Voltage-dependent anion channel (VDAC), (**D**) Carnitine palmitoyl transferase (CPT1), (**E**) ATP synthase 5a (ATP5a) and (**F**) representative images of western blot. β-actin was used as a loading control. One-way analysis of variance (ANOVA), post hoc Fisher´s Least significant difference (LSD), Mean ± SD, n = 3. ^$^ *p* < 0.05 vs. Sham, ^&^ *p* < 0.05 vs. ShamC, ^#^ *p* < 0.05 vs 5/6Nx.

To determine if the observed changes in mitochondria were related to changes in mitochondrial mass and biogenesis, the abundance of PGC1α (Figure 6A,F), PPARα (Figure 6B,F), and VDAC (Figure 6C,F), was measured by WB. PGC1α and PPARα levels were significantly decreased in the 5/6Nx group, but only PPARα was significantly increased in the 5/6NxC group with respect to the 5/6Nx group. VDAC was significantly decreased in the 5/6Nx and 5/6NxC groups with respect to the controls. β-oxidation protein CPT1 was also measured (Figure 6D,F) showing a significant decrease in the 5/6Nx group; the curcumin treatment (5/6NxC) increased it. Furthermore, ATP5a (Figure 6E,F) significantly decreased in the nephrectomy groups compared to the control groups. 

### 3.6. Liver Lipid Metabolism

#### 3.6.1. Liver Free Fatty Acid Profile

Since the liver actively participates in the body´s lipid metabolism, the concentration of free fatty acids in the tissue was measured (Table S2). In addition, total, saturated, unsaturated, and polyunsaturated fatty acids were calculated (Table S2). There were no significant changes in fatty acid liver levels or in total, saturated, unsaturated, and polyunsaturated fatty acids. The C16:1n-7 fatty acid concentration was not detectable in several animals, so C16:1n-7 fatty acid values were not included in Table S2. However, it was considered in the sum of unsaturated and total fatty acids.

#### 3.6.2. Hepatic Levels of Proteins Involved in Lipid Synthesis and Lipogenesis

**Figure 7:**
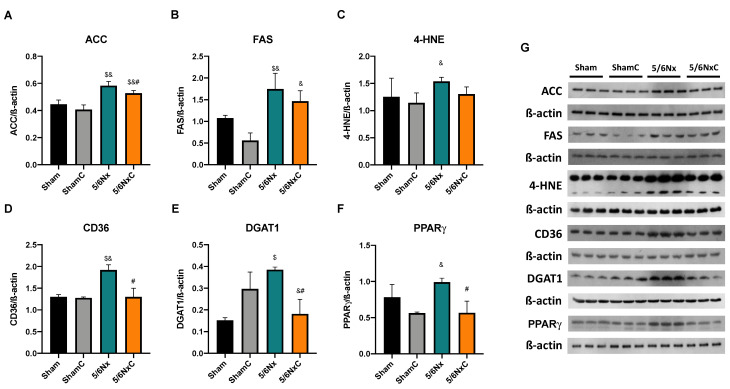
Liver lipid synthesis and storage-related proteins abundance in the four groups of rats studied: Sham, Sham + curcumin (ShamC), five-sixths nephrectomy (5/6Nx), and 5/6Nx + curcumin (5/6NxC). (**A**) Acetyl CoA carboxylase (ACC), (**B**) Fatty acid synthase (FAS), (**C**) 4-hydroxynonenal (4-HNE), (**D**) Fatty acid receptor cluster of differentiation 36 (CD36), (**E**) Diacylglycerol acyl transferase 1 (DGAT1), (**F**) Peroxisome proliferator-activated receptor gamma (PPARγ), and (**G**) representative images of western blot. β-actin was used as a loading control. One-way analysis of variance (ANOVA), post hoc Fisher´s Least significant difference (LSD), Mean ± SD, n = 3. ^$^ *p* < 0.05 vs. Sham, ^&^ *p* < 0.05 vs. ShamC, ^#^ *p* < 0.05 vs. 5/6Nx.

The abundance of ACC and FAS (Figure 7A,B,G) was significantly increased in the 5/6Nx and 5/6NxC groups with respect to the control groups. However, ACC was significantly decreased in the 5/6NxC group compared to the 5/6Nx group. On the other hand, SREBP1 increased its abundance in the 5/6Nx and 5/6NxC groups (Figure S1B). The lipoperoxidation marker (4-HNE) (Figure 7C,G) is increased in 5/6Nx, and treatment with curcumin tends to decrease it. The abundance of CD36, DGAT1 and PPARγ (Figure 7D–G) was significantly increased in the 5/6Nx group, and curcumin treatment significantly decreased it in the 5/6NxC group. 

#### 3.6.3. Liver Mitochondrial β-Oxidation

**Figure 8:**
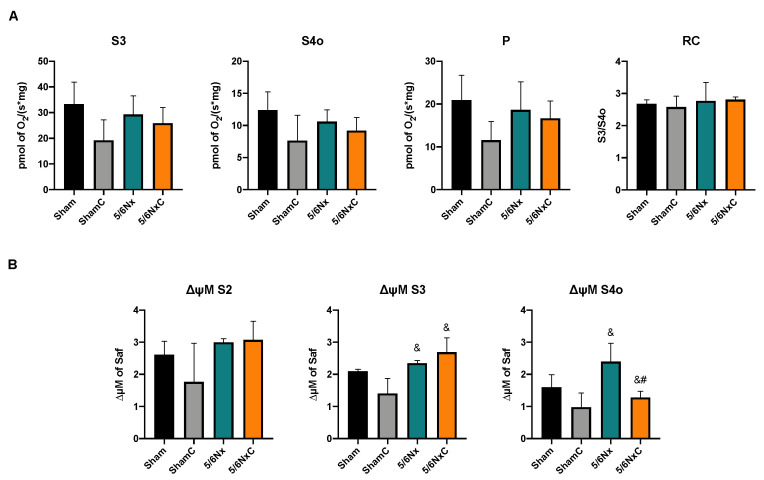
Liver mitochondrial β-oxidation in the four groups of rats studied: Sham, Sham + curcumin (ShamC), five-sixths nephrectomy (5/6Nx), and 5/6Nx + curcumin (5/6NxC). (**A**) Respiration and (**B**) Membrane potential. S3 = State 3, S4o = State 4 induced by oligomycin, P = Oxidative phosphorylation associated respiration, RC = Respiratory control, ΔΨm = Changes in mitochondrial membrane potential, Safranin (Saf). One-way analysis of variance (ANOVA), post hoc Fisher´s Least significant difference LSD, Mean ± SD, n = 3. ^$^ *p* < 0.05 vs. Sham, ^&^ *p* < 0.05 vs. ShamC, ^#^ *p* < 0.05 vs. 5/6Nx.

In the case of the liver, no significant changes were observed between the different groups in the respiration parameters and the ΔΨm when respiration is fed by β-oxidation linked substrates (Figure 8A,B) except for ΔΨm in S4o, which increased in the 5/6Nx group. Curcumin treatment restored the potential of avoiding hyperpolarization in state 4 (Figure 8B).

#### 3.6.4. Hepatic Levels of Proteins Involved in Biogenesis and FA Transport in Mitochondria

**Figure 9:**
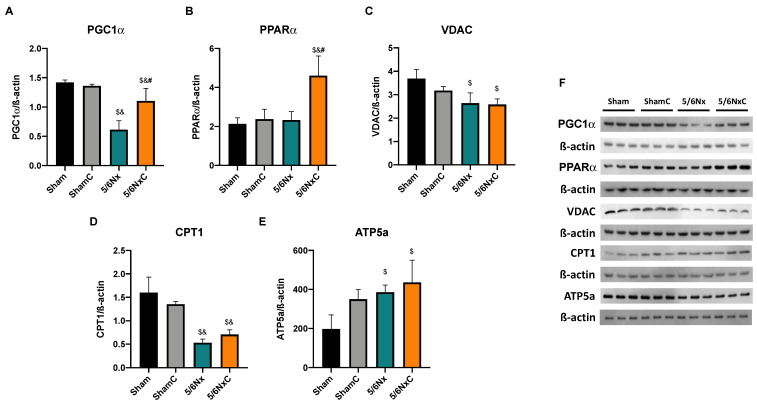
Liver mitochondrial biogenesis and β-oxidation related proteins in the four groups of rats studied: Sham, Sham + curcumin (ShamC), five-sixths nephrectomy (5/6Nx), and 5/6Nx + curcumin (5/6NxC). (**A**) Peroxisome proliferator-activated receptor γ co-activator 1 α (PGC1α), (**B**) Peroxisome proliferator-activated receptor α (PPARα), (**C**) voltage-dependent anion channel (VDAC), (**D**) Palmitoyl carnitine transferase 1 (CPT1), (**E**) ATP synthase 5a (ATP5a) and (**F**) representative images of western blot. β-actin was used as a loading control One-way analysis of variance (ANOVA), post hoc Fisher´s Least significant difference (LSD), Mean ± SD, n = 3. ^$^ *p* < 0.05 vs. Sham, ^&^ *p* < 0.05 vs. ShamC, ^#^ *p* < 0.05 vs. 5/6Nx.

To determine the mitochondrial biogenesis in the liver, we measured the protein levels of PGC1α (Figure 9A,F) and found that it was significantly decreased in the 5/6Nx group compared to the Sham groups, and curcumin treatment increased its abundance significantly. Moreover, the abundance of PPARα (Figure 9B,F) was significantly increased in the 5/6NxC group compared to the other three groups. In order to investigate if the increase in biogenesis was related to mitochondrial mass increase, we evaluated the levels of VDAC, the most abundant outer mitochondrial membrane protein. In the 5/6Nx group, VDAC abundance was significantly less than control groups. The curcumin treatment has no effect (Figure 9C,F). CPT1 was significantly decreased in the 5/6Nx, and 5/6NxC groups (Figure 9D.F), and ATP5a was significantly increased in the 5/6Nx groups with respect to the Sham groups (Figure 9E,F).

## 4. Discussion

Curcumin administration has been widely reported to have nephroprotective effects in experimental models of CKD [32,33,34], [11,35]. Our group previously reported curcumin renal protective effects from 24 h after surgery [34]. Additionally, curcumin reverses glomerular hypertension and oxidative stress in the kidney after 2 months of evolution [11] and promotes tissue regeneration in tubular cells [36]. This is consistent with the reversion of overall parameter alterations (Table 1) and a reduction in markers of kidney damage (Figure 3) and renal TGFβ levels that we observed after curcumin treatment (Figure 2C).

Furthermore, 5/6Nx triggers alterations in other tissues, such as the heart [16], in which curcumin also restores cardiac function [37] and muscle, where there is a loss in muscular mass that curcumin also prevents [38]. This agrees with our results in Table 1, where curcumin reverses the weight loss induced by nephrectomy. Additionally, the increases in food consumption in the 5/6NxC group (Table 1), could be related to the curcumin effect on leptin, an adipokine that produces satiety [39]. In patients with non-alcoholic fatty liver disease, curcumin decreases plasma leptin [40] and reduces leptin gene expression and signaling pathways in cell cultures [41,42]. Therefore, increased food consumption can produce the bioavailability of nutrients that may be used to enhance muscle mass. In this way, our results showed for the first time in the 5/6Nx model that the curcumin protective effect is also present in the liver, where AST and TGFβ levels are also reduced by curcumin (Figure 2E,F) as well as in the histological damage observed with H&E staining (Figure 3L). 

Dyslipidemia is one of the main systemic alterations in patients with CKD, and liver and kidney damage have been closely related. However, the mechanism remains unknown. The alterations observed in the plasma lipid profile and the fatty acids composition in the 5/6Nx group (Table 2 and Table 3) suggest that renal damage is the main factor in dyslipidemia development. Plasma fatty acids must be delivered to the liver to be metabolized, promoting the formation of triglycerides and lipoproteins and their release back into the plasma [43]. Meanwhile, kidney damage reduces mitochondrial lipid use [18], increasing their levels and probably their plasma release, causing hypertriglyceridemia and the lipoprotein plasma increase in 5/6Nx. In CKD, the plasma lipid profile is altered with hypertriglyceridemia, hypercholesterolemia, an increase in VLDL, LDL, and a decrease in HDL [7]. We observed this characteristic profile in the 5/6Nx model except for HDL (Table 2), which increased but could be related to other factors such as its size or lipoprotein oxidation or the fact that we used Wistar rats instead of Sprague–Dawley rats with which the majority of the studies of lipid metabolism in CKD has been performed [14,15,23,24,44]. Curcumin also reversed the 5/6Nx-induced increase in VLDLt, C16:1n-7, and unsaturated fatty acids (Table 2 and Table 3), suggesting kidney and liver protection and partially reversing hyperlipidemia. 

The vast majority of curcumin´s beneficial effects have been associated with its direct and indirect antioxidant capacity [45]. Our group previously reported that curcumin reversed oxidative stress, glomerular hypertension, hyperfiltration, and hemodynamic alterations in 5/6Nx rats [11]. Hemodynamic alterations appear immediately after surgery [46,47], as well as renal perfusion changes and increased oxygen consumption, inducing metabolic shift [48,49,50] and increased ATP demand [34,49]. The lipotoxicity and mitochondrial impairment in the kidneys [51,52,53] triggering metabolic reprogramming is characterized by the shift from mitochondrial-based to anaerobic metabolism, a key mechanism commonly observed in CKD progression [54,55,56]. Nephron sites with a high dependence on mitochondria are among the most affected by the accumulation of lipid deposits [53,57]. Additionally, the elevation in CD36, the primary fatty acid uptake system in the kidney, favors lipid accumulation in CKD models such as the unilateral ureteral obstruction model [9,53,57,58]. This early increase in CD36 facilitates the uptake of long-chain fatty acids and acts as a receptor that triggers inflammatory, oxidative stress, and fibrotic pathways [53,59]. 

Our results agree with the CD36 increase in the 5/6Nx kidney, which together with the rise in FAS and ACC (Figure 4) and the increased lipid deposition (Figure 3D), involves an increase in the uptake and synthesis of lipids by the kidney. Likewise, renal mitochondria in 5/6Nx presented a pathological state characterized by a reduction in OXPHOS capacity and CI and CIII activities, leading to β-oxidation activity reduction from 2 days, which persists [18,34] at week 8 of evolution by the reduction in S3, S4o, P and RC parameters and by lower mitochondrial membrane potential in respiration linked to palmitoyl (Figure 5). Together, the higher lipid uptake carrier levels (Figure 4C) and mitochondria β-oxidation impairment increase acetyl-CoA concentrations that ultimately promote lipid synthesis in the kidney (Figure 4A,B), where lipids accumulate in the parenchyma, favoring inflammation and renal fibrosis. As we observed, the increase in renal fatty acids synthesis enzymes consequently increases the plasma concentration of fatty acids (Table 3), as was previously seen in CKD patients [9]. In addition, the Krebs cycle decreased due to mitochondria damage, increasing acetyl-CoA concentrations and providing more substrates for lipid synthesis [60]. Indeed, kidney biopsies from CKD patients showed reduced mRNA levels of Krebs cycle proteins, which is associated with lower levels of AMP-activated protein kinase (AMPK) and PGC-1α [50,55,61,62,63], the two key regulators of mitochondrial biogenesis. As we observed in Figure 6, at 8 weeks, the decrease in PGC-1α and PPARα leads to the reduction in mitochondrial biogenesis and mass, increasing the kidney´s deterioration. This agrees with previously reported data at 24 h after surgery, where the mitochondrial function is diminished [34]. 

In addition to the effects of curcumin against oxidative stress [18,34], our results showed that curcumin promotes PPARα and CPT1 recovery (Figure 6B,D) as well as oxygen consumption in S4o and the increase in the mitochondrial membrane potential in S3 and S4o (Figure 5A,B). That indicates a partial recovery of mitochondrial β-oxidation in 5/6NxC, which could be associated with the increased activity of the ETS system, as previously demonstrated at 24 h after surgery, where CI activity reduction was prevented [34]. Mitochondrial protection by curcumin in this work (Figure 5 and Figure 6) was lower than previously observed at 24 h. However, we tried a non-preventive treatment, demonstrating the therapeutic effect of curcumin even 8 weeks after surgery. Furthermore, curcumin decreases the CD36 abundance (Figure 4C), reducing kidney fatty acid uptake and synthesis (Figure 4A,B) and, in consequence, lipid accumulation (Figure 3H). These beneficial effects on lipid metabolism and mitochondria can prevent cell damage and, consequently, the formation of collagen fibers (Figure 3G). 

The dyslipidemia of 5/6Nx induces hepatic alterations. Free fatty acids must be transported to the liver to be packed into lipoproteins [64]. Thus, the excessive abundance of fatty acids in the plasma allows their uptake by increased CD36 in the liver (Figure 7D), which induces de novo synthesis characterized by higher ACC, FAS, and DGAT1 levels, promoting PPARγ-mediated lipogenesis. Lipid accumulation (Figure 3K) is also associated with increased oxidative stress, demonstrated by an increase in 4-HNE (Figure 7C), as well as inflammation in this tissue favoring liver damage (Figure 3J). This association has also been described in hepatic steatosis and the non-alcoholic fatty liver disease models [65,66]. Mitochondrial dysfunction is a central axis in the non-alcoholic fatty liver disease pathological process [67]. Therefore, we evaluated liver mitochondrial function in palmitoyl-linked respiration. We observed no changes in respiratory parameters (Figure 8A), but 5/6Nx induces hepatic mitochondrial hyperpolarization (Figure 8B), which can promote mitochondrial ROS overproduction [68], explaining the increased lipid peroxidation (Figure 7C) in the liver. 

**Figure 10:**
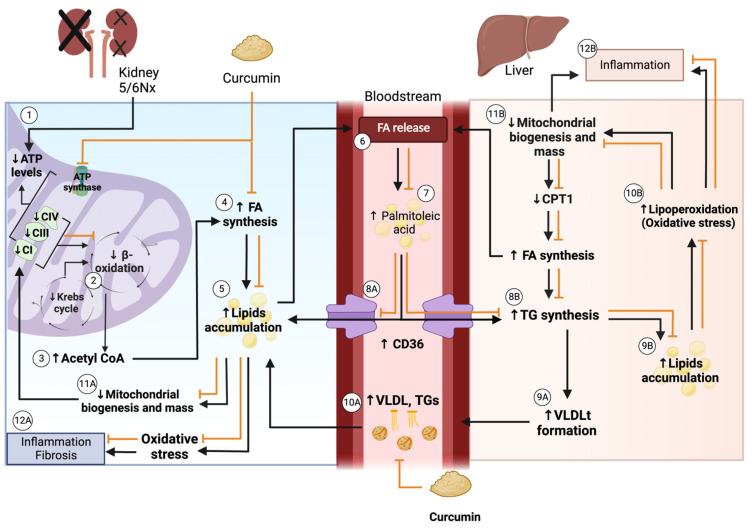
Integrative scheme. (1) In kidney of 5/6 nephrectomy (5/6Nx), adenosine triphosphate (ATP) levels are reduced, attributed to the decrease in the activity of electron transfer system (ETS) elements complex I (CI), CIII, and CIV, and ATP synthase [34]. (2) The decrease in the ETS reduces β-oxidation [18], which along with the reduction in the Krebs cycle, leads to (3) acetyl CoA accumulation [60]. This acetyl CoA is used for (4) fatty acids (FA) synthesis, inducing (5) lipid accumulation, and latter (6) FA release into the bloodstream, particularly (7) palmitoleic acid, which increases in the bloodstream and might (8A) reenter the kidney via a cluster of differentiation 36 (CD36), contributing to lipid accumulation [9]. On the other hand, palmitoleic acid might (8B) enter the liver by CD36, increasing triglycerides (TG) synthesis. TG synthesis increases (9A) the formation of VLDLt, or (9B) contributes to lipid accumulation in the liver. VLDLt proteins (10A) might be delivered in the bloodstream, which increases VLDL and TG levels that could go to the kidney and reinforce lipid accumulation. In the kidney, lipid accumulation (11A) decreases mitochondrial biogenesis and mass, decreasing ETS proteins. Besides, (9B) lipid accumulation in the liver promotes (10B) oxidative stress by inducing lipoperoxidation, which (11B) reduces mitochondrial biogenesis and mass, inducing the decrease in carnitine palmitoyl transferase 1 (CPT1), and later, the increase in FA synthesis, which also induces FA releasing into the bloodstream. Oxidative stress and the reduction in biogenesis and mass contribute to inflammation and fibrotic process (12A) in the kidney and (12B) liver. Curcumin increases the ETS system and β-oxidation in the kidney. It also decreases FA synthesis in the kidney and the liver and these processes could prevent lipid accumulation and, in consequence, the release of significant amounts of FA into the bloodstream. Besides, curcumin decreases CD36 in the kidney and the liver (5/6Nx group), which diminishes FA uptake and lipogenesis in the liver. This could prevent lipoprotein (VLDL) formation in the liver, which was reflected in its bloodstream concentrations that were decreased by curcumin. Thus, curcumin improves mitochondrial function, preventing lipid accumulation in the kidney, liver, and bloodstream and avoiding oxidative stress and tissue injury. The figure was created using BioRender.

Additionally, mitochondrial biogenesis (Figure 9A) and mass markers are decreased (Figure 9C and 9D), in agreement with previous reports where the increase in lipid content decreases liver mitochondrial biogenesis [67]. In contrast, ATP5a levels are increased in the 5/6Nx group. Although we did not explore it deeply, this may be a compensatory response to the observed mitochondrial alteration. Curcumin reverses the increased lipid uptake by CD36 (Figure 7D). It decreases the levels of ACC, DGAT1 and PPARγ (Figure 7A,E.F) that preclude liver lipid accumulation and triglyceride synthesis and consequently decrease the release of VLDL into the bloodstream. Curcumin also increased hepatic levels of the biogenesis proteins PGC1α and PPARα relative to the 5/6Nx group (Figure 9) and reversed hyperpolarization in S4o (Figure 8B), which is associated with the tendency to reduce 4-HNE levels (Figure 7C). Therefore, our results imply that curcumin has beneficial effects on lipid synthesis and mitochondrial metabolism even in the liver. Figure 10 shows an integrative scheme of our data and some taken from the literature.

## 5. Conclusions

The therapeutic effect of curcumin on 5/6Nx hypertriglyceridemia is associated with the restoration of renal mitochondrial ß-oxidation and the reduction in lipid synthesis and uptake in the kidneys and liver.

## Figures and Tables

**Table 1:** General parameters.

Parameter	Groups			
	Sham	ShamC	5/6Nx	5/6NxC
Water consumption (mL/day)	38.42 ± 11.67	62.50 ± 5.93	68.50 ± 7.50 ^$^	72.17 ± 4.80 ^$&^
Food consumption (g/day)	25.92 ± 2.11	26.50 ± 1.60	23.17 ± 1.80 ^$&^	25.25 ± 1.96 ^#^
Body weight (g)	367.30 ± 35.55	397.40 ± 21.00	316.00 ± 24.49 ^$&^	360.40 ± 30.43 ^$&#^

ANOVA, post hoc Tukey, Mean ± SD, *p* < 0.05, n = 7–12. ^$^ *p* < 0.05 vs. Sham, ^&^ *p* < 0.05 vs. ShamC, ^#^ *p* < 0.05 vs. 5/6Nx. ShamC = Sham + curcumin, 5/6Nx: five-sixths nephrectomy 5/6NxC = 5/6Nx + curcumin.

**Table 2:** Plasma lipid profile.

Parameter	Groups			
	Sham	ShamC	5/6Nx	5/6NxC
Triglycerides (mg/dL)	66.44 ± 21.32	56.75 ± 9.824	84.63 ± 20.66 ^&^	63.62 ± 9.43 ^#^
Cholesterol (mg/dL)	43.77 ± 4.574	42.43 ± 6.867	87.28 ± 14.99 ^$&^	79.54 ± 13.19 ^$&^
HDLc (mg/dL)	30.48 ± 4.523	29.09 ± 3.555	61.2 ± 9.517 ^$&^	59.62 ± 10.55 ^$&^
LDLc (mg/dL)	11.16 ± 2.363	11.44 ± 3.863	23.65 ± 5.918 ^$&^	22.58 ± 6.578 ^$&^
VLDLc (mg/dL)	2.122 ± 1.91	2.334 ± 1.98	4.267 ± 3.805	5.263 ± 7.382
VLDLt (mg/dL)	11.36 ± 5.377	11.35 ± 1.963	16.92 ± 4.133 ^&^	12.72 ± 3.886 ^#^

ANOVA, post hoc Tukey, Mean ± SD, *p* < 0.05, n = 7–12. ^$^ *p* < 0.05 vs Sham, ^&^ *p* < 0.05 vs. ShamC, ^#^ *p* < 0.05 vs. 5/6Nx. ShamC = Sham + curcumin, 5/6Nx: five-sixths nephrectomy 5/6NxC = 5/6Nx + curcumin, HDLc = High-density lipoproteins cholesterol, LDLc = Low-density lipoproteins cholesterol VLDLc = Very low-density lipoproteins cholesterol VLDLt = Very low-density lipoproteins triglycerides.

**Table 3:** Plasma fatty acids concentration.

Fatty Acids (mol/L)	Groups			
	Sham	ShamC	5/6Nx	5/6NxC
Lauric acid (C12)	0.005 ± 0.009	0.006 ± 0.002	0.004 ± 0.001	0.003 ± 0.001 ^&^
Miristic acid (C14)	0.004 ± 0.002	0.004 ± 0.002	0.006 ± 0.003	0.004 ± 0.001
Palmitic acid (C16)	0.119 ± 0.039	0.113 ± 0.011	0.193 ± 0.046 ^&^	0.169 ± 0.023 ^$&^
Palmitoleic acid (C16:1n-7)	0.002 ± 0.001	0.004 ± 0.002	0.012 ± 0.002 ^$&^	0.006 ± 0.002 ^$#^
Estearic acid (C18)	0.057 ± 0.014	0.052 ± 0.006	0.093 ± 0.019 ^$&^	0.088 ± 0.018 ^$&^
Oleic acid (C18:1n-9)	0.054 ± 0.028	0.050 ± 0.010	0.091 ± 0.036 ^&^	0.076 ± 0.019
Linoleic acid (C18:2n-6)	0.083 ± 0.034	0.079 ± 0.015	0.160 ± 0.037 ^$&^	0.125 ± 0.022 ^&^
α-Linolenic acid (C18:3n-3)	0.002 ± 0.001	0.002 ± 0.001	0.004 ± 0.002	0.002 ± 0.001
γ-Linolenic acid (C18:3n-6)	0.001 ± 0.0004	0.002 ± 0.0003	0.002 ± 0.001	0.002 ± 0.0003
Dihomo-gamma-linolenic acid (C20:3n-6)	0.001 ± 0.0007	0.002 ± 0.0009	0.002 ± 0.0009	0.002 ± 0.0002
Arachidonic acid (C20)	0.068 ± 0.012	0.049 ± 0.008	0.133 ± 0.029 ^$&^	0.099 ± 0.029 ^&^
Saturated fatty acids	0.210 ± 0.058	0.180 ± 0.022	0.299 ± 0.068 ^$&^	0.255 ± 0.039 ^&^
Unsaturated fatty acids	0.063 ± 0.029	0.051 ± 0.013	0.115 ± 0.031 ^$&^	0.075 ± 0.011 ^#^
Polyunsaturated fatty acids	0.093 ± 0.038	0.087 ± 0.013	0.169 ± 0.040 ^$&^	0.129 ± 0.023
Total	0.419 ± 0.151	0.378 ± 0.056	0.680 ± 0.182 ^$&^	0.552 ± 0.082

ANOVA, post hoc Tukey, Mean ± SD, *p* < 0.05, n = 6–8. ^$^ *p* < 0.05 v.s Sham, ^&^ *p* < 0.05 vs. ShamC, ^#^ *p* < 0.05 vs. 5/6Nx. ShamC = Sham + curcumin, 5/6Nx: five-sixths nephrectomy 5/6NxC = 5/6Nx + curcumin.

## Data Availability

The data are contained within this article.

## Supplementary Materials

The following supporting information can be downloaded at: https://www.mdpi.com/article/10.3390/antiox11112195/s1.
