# Supplementary material for: Therapeutic Effect of Curcumin on 5/6Nx Hypertriglyceridemia: Association with the Improvement of Renal Mitochondrial β-Oxidation and Lipid Metabolism in Kidney and Liver
Source: Antioxidants (Basel). 2022 Nov 6;11(11):2195. doi: 10.3390/antiox11112195 (PMC9686550; doi:10.3390/antiox11112195)
Supplement: Supplementary file 1 [file antioxidants-11-02195-s001.zip › antioxidants-1996390-supplementary.pdf]

Suplementary Material.

Table S1. Kidney fatty acid profile

	Groups			
**Fatty acids (10^{-5} mol/µg protein)**	**Sham**	**ShamC**	**5/6Nx**	5/6Nx**C**
**Lauric acid (C12)**	6.280±2.848	4.260±1.115	6.3±1.804	8.640±1.110$^{\&}$
**Miristic acid (C14)**	4.467±1.343	3.717±0.933	4.767±2.196	3.767±1.381
**Palmitic acid (C16)**	1.175±2.620	1.020±1.457	1.337±2.673	1.380±2.665
**Palmitoleic acid (C16:1n-7)***	0.567±0.252	0.560±0.568	1.525±0.395$^{\$\&}$	1.667±0.321$^{\$\&}$
**Estearic acid (C18)**	1.117±0.584	0.833±0.250	1.383±1.053	1.060±0.416
**Oleic acid (C18:1n-9)**	9.917±2.356	8.317±1.459	9.4±2.428	9.5±2
**Linoleic acid (C18:2n-6)**	3.7±2.067	3.033±0.753	3.5±0.743	2.767±0.432
**α-Linolenic acid (C18:3n-3)**	3.2±0.754	2.75±0.554	3.033±0.829	2.617±0.542
**γ-Linolenic acid (C18:3n-6)**	2.85±1.268	2.767±0.956	3.317±2.101	3.483±1.202
**Dihomo-gamma-linolenic acid (C20:3n-6)**	0.717±0.248	0.6±0.276	0.967±0.635	1.333±0.650
**Arachidonic acid (C20)**	2.8±1.121	2.733±1.021	2.633±2.294	2.467±0.859
**Saturated fatty acids**	24.80±4.817	20.33±3.077	30.20±3.701$^{\&}$	26.20±1.789
**Unsaturated fatty acids**	10.20±2.224	8.783±1.656	10.40±2.418	10.33±2.194
**Polyunsaturated fatty acids**	10.67±2.805	9.167±1.549	10.80±3.461	10.20±1.251
**Total**	46.18±4.650	40.33±4.473	53.60±6.791$^{\&}$	47.34±2.871

ANOVA, post hoc Tukey or Fishers LSD. Mean SD, p<0.05, n=3-6. $p<0.05 vs Sham, &p<0.05 vs ShamC. ShamC = Sham+curcumin, 5/6Nx: five sixths nephrectomy 5/6NxC=5/6Nx+curcumin

Table S2. Liver fatty acids profile.

	Groups			
**Fatty acids (10^{-5} mol/µg protein)**	**Sham**	**ShamC**	**5/6Nx**	**5/6NxC**
**Lauric acid (C12)**	0.823±0.499	0.461±0.114	0.631±0.405	0.891±0.521
**Miristic acid (C14)**	5.071±4.794	0.866±0.438	1.996±1.353	4.303±3.467
**Palmitic acid (C16)**	17.54±5.581	12.88±2.085	11.45±2.860	14.71±4.156
**Estearic acid (C18)**	11.44±3.837	8.314±1.366	7.903±3.519	9.692±2.272
**Oleic acid (C18:1n-9)**	2.911±1.269	2.389±0.369	2.101±0.413	2.911±1.287
**Linoleic acid (C18:2n-6)**	6.451±3.601	5.323±4.986	2.445±0.979	3.450±1.193
**α-Linolenic acid (C18:3n-3)***	1.658±0.529	2.348±1.568	1.475±0.668	1.520±1.191
**γ-Linolenic acid (C18:3n-6)***	1.812±0.731	1.582±1.551	1.459±0.897	1.283±1.717
**Dihomo-gamma-linolenic acid (C20:3n-6)**	2.915±0.804	2.371±0.310	1.990±0.593	2.908±0.624
**Arachidonic acid (C20)**	5.020±4.336	15.03±28.23	2.424±1.063	3.395±2.535
**Saturated fatty acids**	38.92±17.41	37.33±30.11	23.90±7.508	32.31±8.525
**Unsaturated fatty acids**	3.009±1.338	6.222±8.988	2.170±0.489	3.023±1.384
**Polyunsaturated fatty acids**	11.10±4.503	10.97±6.664	5.902±2.319	8.018±3.962
**Total**	53.03±19.90	54.52±45.19	31.97±7.162	43.35±10.40

ANOVA, post hoc Tukey or Fishers LSD. Mean SD, p<0.05, n=3-6. $p<0.05 vs Sham, &p<0.05 vs ShamC. ShamC = Sham+curcumin, 5/6Nx: five sixths nephrectomy 5/6NxC=5/6Nx+curcumin

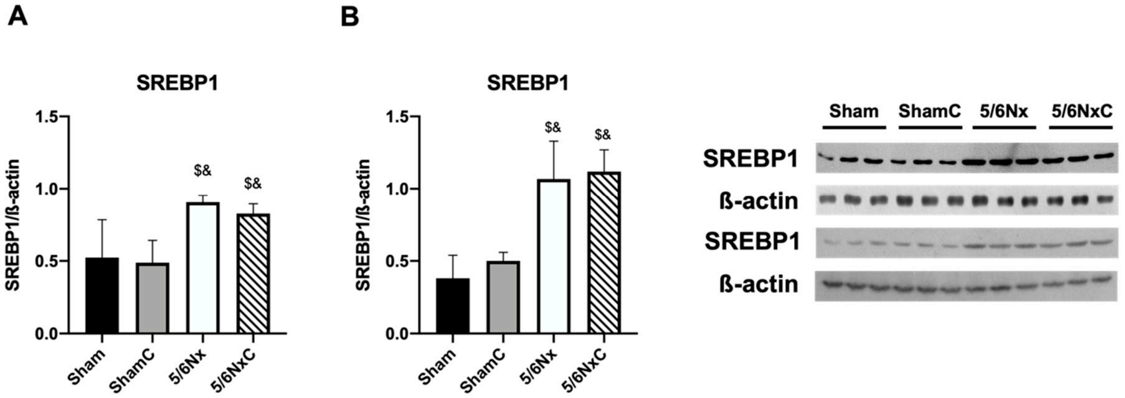

Figure S1. Sterol receptor element binding protein 1, A) Kidney, B) Liver . β-actin was used as a loading control One way analysis of variance (ANOVA), post hoc Fisher´s Least significant difference (LSD), Mean±SD, n=3. $ p<0.05 vs Sham, & p<0.05 vs ShamC, # p<0.05 vs 5/6Nx
